# A Novel 5-Chloro-*N*-Phenyl-1 *H*-Indole-2-carboxamide Derivative as a Glycogen Phosphorylase Inhibitor: Evaluating the Long-Term Drug Effects on Muscle Function for the First Time

**DOI:** 10.3390/molecules29184448

**Published:** 2024-09-19

**Authors:** Yifan Zhao, Zhiwei Yan, Shuai Li, Youde Wang, Yachun Guo, Tienan Wang, Liying Zhang

**Affiliations:** 1 Laboratory of Traditional Chinese Medicine Research and Development of Hebei Province, Institute of Traditional Chinese Medicine, Chengde Medical University, Chengde 067000, China; 13379341279@163.com (Y.Z.); cdyanzhiwei@163.com (Z.Y.); cmuyhls@163.com (S.L.); wangyoude8686@126.com (Y.W.); cdwangtienan@163.com (T.W.); 2 Department of Pathogen Biology, Chengde Medical University, Chengde 067000, China; gyc123263295@126.com

**Keywords:** diabetes, GP inhibitors, muscle function, muscle energy metabolism, adverse effects

## Abstract

Compound **1** was previously identified by our team as a glycogen phosphorylase (GP) inhibitor with glucose-lowering activity and demonstrated to have protective effects against myocardial and cerebral ischemia. However, its impact on muscle function has not been clarified. This study is the first to evaluate the long-term effects of GP inhibitors on muscle function and metabolism. After a 28-day administration of Compound **1**, we performed assays to assess muscle function and biochemical parameters in rats. We observed reductions in peak holding force, duration, tetanic contraction force, single-contraction force, and electromyographic signals under 20 s and 10 min contraction stimuli. The metabolic analysis showed no significant effects on muscle glycogen, ATP, lactic acid, and uric acid levels at low doses. In contrast, medium to high doses resulted in increased glycogen, decreased ATP, and reduced lactic acid (only at high doses), without affecting uric acid. These findings suggest that Compound **1** may adversely affect muscle function in rats, potentially due to the glycogen inhibition effects of GP inhibitors. This study provides crucial safety data and insights into the long-term effects of GP inhibitors on rat muscles, which will guide future developments and applications.

## 1. Introduction

The *IDF Diabetes Atlas 10th edition* reported that approximately 537 million people worldwide have diabetes, indicating that diabetes has become a significant threat to human health [1]. The challenge of long-term glucose control with single-mechanism drugs may lead to treatment failure due to the complex etiology of diabetes, underscoring the clinical need for new targeted glucose-lowering therapies [2]. Glycogen phosphorylase (GP) catalyzes the breakdown of glycogen into glucose in the liver and other tissues to meet energy demands [3,4]. Glycogenolysis and glycogenesis are essential processes for regulating blood glucose levels. GP could be a viable target for diabetes treatment, making GP inhibitors a potential avenue for developing new glucose-lowering drugs [5].

**Figure 1:**
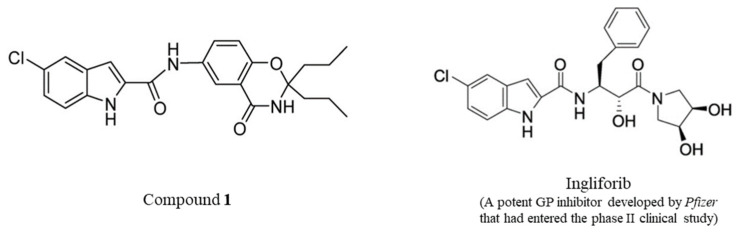
The structures of Compound **1** and Ingliforib.

Additionally, there is increasing interest in developing glucose-lowering drugs with therapeutic effects beyond glucose control, such as cardiovascular benefits, which have been highlighted in recent guidelines [6,7,8]. GP inhibitors, like Ingliforib (Figure 1), developed by Pfizer, have shown promise in animal studies, with cardioprotective effects [9,10]. Similarly, Compound **1** (Figure 1), previously reported as a GP inhibitor with glucose-lowering activity, has demonstrated cardiovascular benefits [11,12,13,14,15]. This underscores the potential of GP inhibitors as novel glucose-lowering drugs for diabetes treatment.

However, these antidiabetic medications have not progressed to the clinical phase, primarily due to challenges in targeting specific GP isoforms. This difficulty can lead to insufficient glycogen in skeletal muscle, adversely affecting muscle function [10]. Given that exercise and physical training are fundamental to diabetes management, further research is needed to explore the effects of GP inhibitors on muscle function [8].

Short-term trials have shown that Ingliforib appears to have a minimal impact on muscle function; however, no long-term trials have been conducted [9]. Given that diabetes treatment often requires long-term management [16], this study aimed to evaluate the long-term effects of GP inhibitors on muscle function for the first time.

We selected Ingliforib as the positive control and determined the effective doses of Compound **1** and Ingliforib based on previous studies [9,15]. In this study, we administered Compound **1** or Ingliforib to rats via tail vein injection for 28 days, assessed muscle function using the rats’ hindlimbs, and analyzed muscle metabolism through biological assays. We confirmed that the long-term administration of both GP inhibitors adversely affected muscle function in rats and explored potential causes of this effect. Our findings aim to guide the development and application of GP inhibitors.

## 2. Result and Discussion

### 2.1. Effects of Compound ***1*** on Muscle Function in Rat

#### 2.1.1. The Impact of Compound **1** on Muscle Grip Strength in Rats

**Figure 2:**
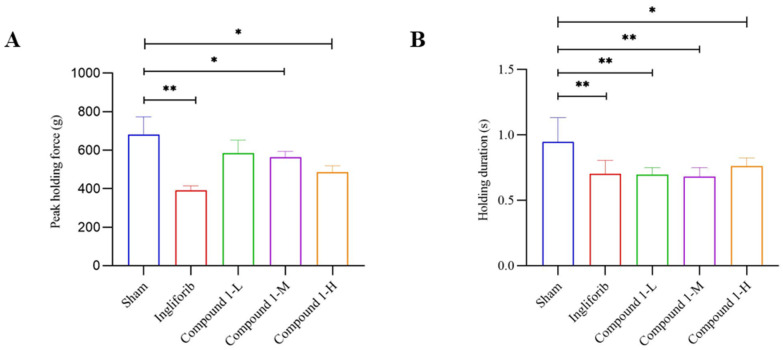
The impact of Compound **1** on peak holding force (**A**) and holding duration (**B**) in rats. * *p* < 0.05; ** *p* < 0.01.

We used a grip strength meter to measure the grip strength and duration of rats to assess the long-term impact of Compound **1** on muscle function [17]. The groups were categorized as Sham, Ingliforib, and Low-, Medium-, and High-dose groups of Compound **1** (Low, 1.25 mg/kg; Medium, 2.5 mg/kg; High, 5 mg/kg). The results for Compound **1** and Ingliforib after 28 days of tail vein injection are shown in Figure 2. Compared to the Sham group, the muscle peak holding force was significantly reduced in the Ingliforib group and the Medium- and High-dose groups of Compound **1**. However, a low dose of Compound **1** did not affect the peak holding force. Regarding holding duration, both the Compound **1** and Ingliforib groups exhibited significantly reduced durations compared to the Sham group. These results indicate that the long-term administration of high and medium doses of Compound **1** reduced muscle peak holding force in rats, suggesting a potential adverse effect of Compound **1**. Additionally, the reduction in holding duration observed in both the Compound **1** and Ingliforib groups may reflect another adverse effect, possibly specific to GP inhibitors, on muscle function.

#### 2.1.2. The Impact of Compound **1** on the Mechanical Properties of Load-Bearing Muscles in Rats’ Hindlimbs

**Figure 3:**
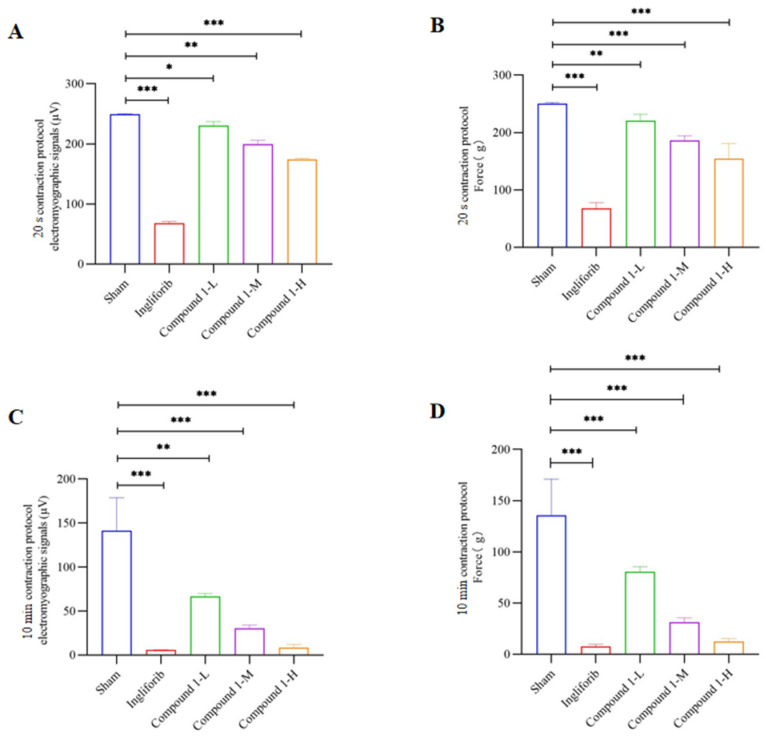
The impact of Compound **1** on the mechanical properties of load-bearing muscles in rats’ hindlimbs. Emg signals (**A**) and muscle tone (**B**) in the 20 s contraction protocol; Emg signals (**C**) and muscle tone (**D**) in the 10 min contraction protocol. * *p* < 0.05; ** *p* < 0.01; *** *p* < 0.001.

To explore the impact of Compound **1** on the mechanical properties of load-bearing muscles in rats’ hindlimbs, we employed an experimental protocol involving 20 s maximum isometric contractions (30 Hz, 200 ms, 10 V) and 10 min intermittent maximum isometric contractions (30 Hz, 200 ms, 10 V) to assess changes in the contraction strength and electromyographic signals of rat hindlimb muscles [17]. The results from the 20 s contraction protocol are shown in Figure 3A,B. Compared to the Sham group, the isometric tetanic force and electromyographic signals in the hindlimbs of rats in the Ingliforib group and the different dose groups of Compound **1** were significantly reduced. The results from the 10 min contraction protocol, depicted in Figure 3C,D, showed varying degrees of decline in single-contraction force and electromyographic signals in the Ingliforib group and the different dose groups of Compound **1**. The decrease in single-contraction force was less pronounced in the Low-dose Compound **1** group compared to the Ingliforib group.

These results indicate that both Compound **1** and Ingliforib lead to significant decreases in tetanic and single-contraction forces, as well as in the electromyographic signals of rats’ hindlimb muscles. Additionally, the effects of Compound **1** on tetanic and single-contraction forces and electromyographic signals in the rat hindlimb are dose-dependent.

### 2.2. The Impact of Compound ***1*** on Rats’ Muscle Metabolism

#### 2.2.1. The Effect of Compound **1** on the Glycogen Content in Rat Muscles

**Figure 4:**
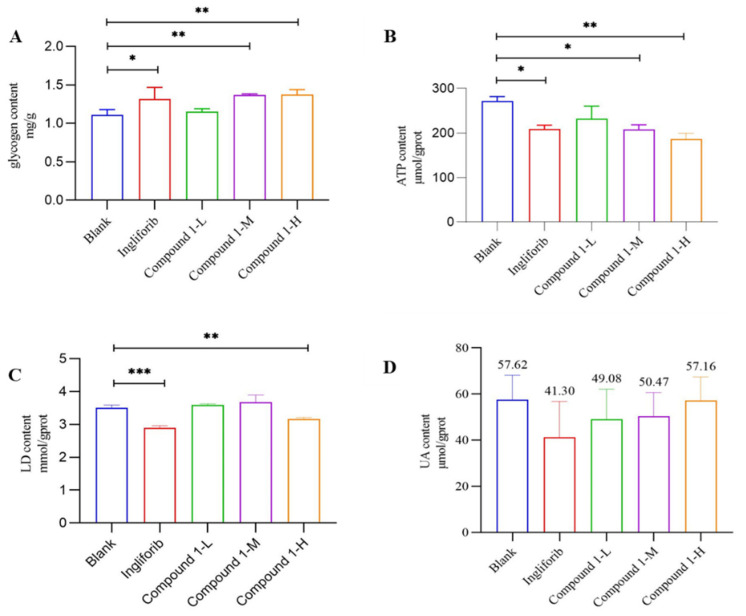
The impact of Compound **1** on rats’ muscle metabolism: (**A**) glycogen content; (**B**) ATP content; (**C**) LD content; (**D**) UA content. * *p* < 0.05; ** *p* < 0.01; *** *p* < 0.001.

Glycogen serves as an energy source for muscle tissue. Under the action of glycogen phosphorylase, it is broken down into glucose, which provides energy for muscle activity [18,19]. Therefore, we measured the glycogen content after continuous injection over 28 days. The results, shown in Figure 4A, reveal a significant increase in muscle glycogen content in both the Medium- and High-dose groups of Compound **1** and the Ingliforib group compared to the Blank group. However, no significant difference in muscle glycogen content was observed between the Low-dose group of Compound **1** and the Blank group. This suggests that the long-term administration of Compound **1** or Ingliforib affects muscle glycogen metabolism, potentially due to glycogen accumulation resulting from glycogen phosphorylase inhibition. Therefore, Compound **1** may impact the glycogen content in rats’ muscles, thereby influencing the energy metabolism.

#### 2.2.2. The Impact of Compound **1** on Rat Muscles’ Energy Metabolism

ATP plays a crucial role in muscle activity by providing energy [20], and it is produced through glycolysis, which is closely related to lactic acid (LD) [21]. To explore the effects of Compound **1** on muscle energy metabolism, we measured the ATP content in rat muscle tissue after 28 days of continuous injection. The results, shown in Figure 4B, indicate a significant decrease in ATP content in both the Medium- and High-dose groups of Compound **1** and the Ingliforib group compared to the Blank group. No significant difference in ATP content was observed between the Low-dose group of Compound **1** and the Blank group.

Glucose produced from glycogen breakdown is a key source of ATP. Our findings suggest that Compound **1** and Ingliforib may inhibit glycogen breakdown, leading to a reduction in ATP sources and, consequently, a decrease in ATP content. This observation supports the effects seen in muscle tissue. Therefore, the long-term administration of Compound **1** may decrease the ATP content in muscles, potentially impacting muscle function.

#### 2.2.3. The Impact of Compound **1** on LD Release in Rat Muscles

LD is an important intermediate product in energy generation and is widely present in various tissues, including skeletal muscle [22,23]. The measurement results for LD content, shown in Figure 4C, only reveal a significant reduction in LD content in the High-dose group of Compound **1** and the Ingliforib group compared to the Blank group. The LD content is related to glycolysis, as LD is produced during this process. The inhibition of glycolysis leads to decreased LD content. Thus, the reduction in lactate content observed with long-term administration of Compound **1** or Ingliforib may be attributed to their GP inhibitory properties.

#### 2.2.4. The Effect of Compound **1** on Uric Acid in Muscles

Uric acid (UA) is the terminal product of purine metabolism. During high-intensity or anaerobic exercise, ATP in muscle cells undergoes extensive hydrolysis to provide energy, yielding adenosine and hypoxanthine as intermediates, which are subsequently metabolized to generate UA [24]. To elucidate the impact of Compound **1** on the metabolic processes of rat muscle, we assayed the UA concentration in rat muscle tissue. The analysis, shown in Figure 4D, indicates that Compound **1** and Ingliforib did not significantly alter the UA levels in muscles compared to the control group. However, it is worth noting that the absolute UA value for Ingliforib appears to trend downward compared to the Blank group (Blank vs. Ingliforib, 57.62 ± 16.3 vs. 41.30 ± 8.96 μmol/g protein, *p* = 0.21). Therefore, the long-term administration of Compound **1** or Ingliforib does not significantly affect UA levels in muscles.

## 3. Conclusions

Our experiments demonstrate that the long-term use of Compound **1**, under both 20 s and 10 min contraction stimuli, leads to a decrease in peak grip strength and grip duration in rat muscles. Additionally, it results in a significant reduction in the tetanic and single-contraction electromyographic signals of the rats’ hindlimbs. Further analysis delineated the impact of Compound **1** on rats’ muscle metabolism. It was observed that a low dose of Compound **1** did not alter the levels of glycogen, ATP, LD, or UA in rat muscles after 28 days of injection. In contrast, medium to high doses of Compound **1** resulted in elevated glycogen levels, reduced ATP levels, and a decrease in LD levels (especially at the high dose), while the UA levels remained unaffected. These findings suggest that the long-term administration of Compound **1** adversely affects muscle function in rats, with Ingliforib showing similar effects. Thus, these effects may be related to the glycogen-inhibitory properties of GP inhibitors.

In conclusion, this study reveals that the adverse effects of long-term use of GP inhibitors on muscle function may be an inherent property of these inhibitors and explores the mechanisms underlying these effects. This provides new safety data and valuable references for the future development and application of GP inhibitors.

## 4. Materials and Methods

### 4.1. Animals

Male SPF SD rats were obtained from Beijing Huafukang Biotechnology Co., Ltd. (Beijing, China). All animal studies, including euthanasia procedures, were conducted in compliance with the regulations and guidelines of Chengde Medical University’s institutional animal care program and adhered to the standards set by the Association for the Assessment and Accreditation of Laboratory Animal Care International (AAALAC) and the Institutional Animal Care and Use Committee (IACUC).

### 4.2. Injection Material Configuration Method

Compound **1** and Ingliforib were dissolved in DMSO (10%) and mixed with PEG300 (40%), and then, Tween 80 (5%) was added. Saline was then added to adjust the final volume to 45%.

### 4.3. Experiment Grouping

Sixty SPF SD rats, with an average body weight of 200–220 g, were randomly and evenly divided into five groups, with twelve rats per group:

Blank/Sham Group (no medication was administered);
Compound **1** groups (1.25 mg/kg, 2.5 mg/kg, 5 mg/kg);
Ingliforib group (30.4 mg/kg).

Following grouping, the medications were administered via tail vein injection for 28 days.

Note: Blank group represents the biological experimental group; Sham Group represents muscle experimental group.

### 4.4. Muscle Grip Strength Testing

The grip strength of rat was assessed using a grip strength meter designed for small and medium-sized animals. Once the device was activated, the rat was placed on the grip platform. The tail of the rat was gently pulled backward while it was held until the animal firmly grasped the platform. Even force was then applied to pull the rat backward until it released its grip. At this point, the device automatically recorded the maximum grip strength and the duration of the grip. Each rat underwent three trials of grip strength testing, with a minimum interval of 5 min between each test.

### 4.5. Muscle Contraction and Electromyography Signal Testing

After completing the grip strength test, rats were thoroughly anesthetized. The skin of the left hind limb was then removed, and the branches of the femoral artery and vein were ligated until the femoral artery entered the gastrocnemius muscle in the lower leg. The biceps femoris muscle was removed, and the distal end of the Achilles tendon was ligated and cut to ensure that the gastrocnemius muscle remained attached to the back of the knee joint. Following this, the rats were euthanized, and their tibiae were secured in clamps on a three-dimensional frame. The Achilles tendon was connected to an isometric force transducer. During the measurement of contractile muscle force, the tibia was fixed to minimize inertia caused by animal movement. Contractile tension and electromyographic signals were recorded to assess muscle contraction.

Muscle contraction was assessed as follows, with each group divided into three subgroups based on different treatments:

1. Immediate excision of the gastrocnemius–plantaris–soleus(GPS) group and immediate freezing with liquid nitrogen;
2. A 20 s maximum isometric contraction intensity (30 Hz, 200 ms, 10 V);
3. A 10 min submaximal isometric contraction intensity (1 Hz, 0.3 ms, 2 V).

Muscle contraction was induced by direct electrical stimulation of the sciatic nerve using hook electrodes, with the isometric tension recorded throughout the process. Arterial and venous blood samples were collected every 2 min during rest and throughout the 10 min submaximal intensity contraction to determine the perfused lactate concentration.

### 4.6. Muscle Metabolism Detection

Immediately following the contraction test, the GPS muscle group was swiftly excised and rapidly frozen in liquid nitrogen. The muscle group was weighed, placed into liquid nitrogen for analysis, crushed in liquid nitrogen, and thoroughly mixed. It was then freeze-dried overnight at −80 °C. The glycogen, ATP, and uric acid concentrations in the muscle were determined using ELISA [25], while the muscle lactate concentrations were measured using spectrophotometry [26,27].

### 4.7. Materials

Rat Grip Strength Meter, Xinruan Biotechnology Co., Ltd. (Shanghai, China); Multiskan FC Enzyme labeled analyzer, Thermo Fisher (Waltham, MA, USA); Implantable electromyography electrodes, A-M Systems (Sequim, WA, USA); EDTA Antigen Retrieval Solution, Guge Biotechnology Co., Ltd. (Wuhan, China); binocular operating microscope, XTX-4A, Zhenjiang Xintian Medical Instruments Co., Ltd.(Zhejiang, China); surgical instruments, World Precision Instruments, Inc. (Sarasota, FL, USA); general anesthesia machine for small animals R500, Ruiwo Life Technology Co., Ltd. (Shenzhen, China); biological signal acquisition and processing system, MedLab.U/8C502, Meiyi Technology Co., Ltd. (Nanjing, China); Ethanol and Xylene, Guanghua Technology Co., Ltd. (Shantou, China); Formaldehyde solution and Hydrogen peroxide solution, Hengjian Pharmaceutical Co., Ltd. (jiangmen, China); glycogen content test kit, Solarbio Biotechnology Co., Ltd. (Beijing, China); LD content test kit, Solarbio Biotechnology Co., Ltd. (Beijing, China); uric acid (UA) Colorimetric Assay Kit, Elabscience Biotechnology Co., Ltd. (Wuhan, China); ATP Assay Kit, Bevotime (Shanghai, China); Ingliforib, Huiyou Biotechnology Co., Ltd. (Shijiazhuang, China), with a purity of 99.64%; Compound **1** was synthesized and mass-produced by our team, with a purity of 99.32%; DMSO, PEG300, Tween-80, and Saline, Merck KGaA (Darmstadt, Germany).

### 4.8. Statistical Analysis

Data are expressed as the mean ± SD. Comparisons between the experimental and control groups were conducted using Student’s *t*-test with nonparametric data. Statistical significance was set at *p* < 0.05.

## Data Availability

The data presented in this study are available on request from the corresponding author.
